# Preliminary Study on Functional and Aesthetic Reconstruction by Using a Small Artery-only Free Medial Flap of the Second Toe for Fingertip Injuries

**DOI:** 10.6061/clinics/2019/e1226

**Published:** 2019-10-14

**Authors:** Muwei Li, Ming Huang, Yanjun Yang, Liqiang Gu, Ziqing Zhang, Yi Yang

**Affiliations:** I Department of Microsurgery and Hand Surgery, the Long gang Orthopedics Hospital of Shen zhen, Shen zhen, 518116, China; II Zhong shan School of Medicine, Sun Yat-sen University, Guang zhou, 510080, China.; III Department of Microsurgery and Orthopedic Trauma, the First Affiliated Hospital of Sun Yat-sen University, Guang zhou, 510080, China

**Keywords:** Artery-Only, Free Medial Flap of Second Toe, Fingertip Injuries

## Abstract

**OBJECTIVES:**

This study was designed to introduce the feasibility of fingertip reconstruction by using a free medial flap of the second toe without vein anastomosis.

**METHODS:**

In total, 8 patients with fingertip injuries were treated successfully with this method. Patients who underwent reconstruction from September 2016 to October 2017 in our hospital with an artery-only free medial flap transfer of the second toe for fingertip injuries were included, and patients who underwent additional procedures that may impact the postoperative results and were followed up for less than 6 months were excluded. Clinical trial registration: ChiCTR19000021883.

**RESULTS:**

According to the Allen classification, five patients had Type 3 injuries, and three patients had Type 4 injuries. One arterial nerve and one digital nerve were repaired at the same time. No additional dissection was performed in either the donor or recipient site of the dorsal or volar vein. Postoperative venous congestion was monitored based on the color, temperature and the degree of tissue oxygen saturation. The flap size ranged from 1.20*1.0 cm^2^ to 1.80*1.0 cm^2^. The reconstruction time was 71.86 (SD 14.75) minutes. The two-point discrimination and the monofilament results were satisfying; cold intolerance did not appear in five patients, and the other three patients had cold intolerance with grades of 4, 12 and 26, which were considered satisfactory. Moreover, leech therapy, continuous bleeding and needle sutures were not utilized in any cases.

**CONCLUSIONS:**

Reconstruction with a small artery-only free medial flap transfer of the second toe led to satisfactory sensory and motor function in the selected patients with fingertip injuries.

## INTRODUCTION

The fingertip is defined as the part of the finger between the distal point and the insertion of the flexor and extensor tendons. Due to its specialized anatomy and unique structure, the fingertip plays an important role in essential functions such as gripping, agreeable handling and sensing in daily life (1).

Fingertip reconstruction techniques, such as V-Y advancement flaps, pedicle flaps, and free flaps, have been improved tremendously to meet functional and aesthetic demands. Nevertheless, challenges still exist, and fingertip replantation is not always possible due to influential factors including ischemia time and availability of surgical resources (2,3); in addition, amputation with wound closure may lead to problems such as hook-nail deformity or neuroma formation (4) or the inability to perform vein anastomosis in free flap (such as the free medial flap of the second toe) transfer during fingertip reconstruction due to a vein shortage in the donor or the recipient site (5).

This study introduces a novel technique for reconstruction using the artery-only free medial flap of the second toe for fingertip injuries.

## METHODS

### Ethical standards and Statement of Consent

This research protocol was approved in advance by the appropriate ethical committee of Sun Yat-sen University and the Long Gang Orthopedics Hospital, and informed consent was provided by all patients. All of our methods were carried out in accordance with approved guidelines. The Clinical Trial registration number is ChiCTR1900021883.

### Patients

The preliminary diagnosis was made on the basis of a detailed history and examination. Patients suffering from Type 3 or 4 injuries, according to the Allen classification, who underwent reconstruction from September 2016 to October 2017 in our hospital with the artery-only free medial flap of the second toe for fingertip injuries were included. Patients who underwent additional procedures that may impact the postoperative results and were followed up for less than 6 months were excluded. In addition, patients suffering from high blood pressure, diabetes, smoking addiction or vascular disease were also excluded from this study. All surgeries and postoperative management were performed by the same team. Furthermore, patient age, sex, etiology, follow-up times, complications and outcomes were all reported. In addition, in the first stage, the patients’ injured fingertips were irrigated and debrided, and vacuum-assisted closure (VAC) devices were applied to cover the wounds within 8 hours after injury. Then, in the second stage, the fingertip defects were covered by an artery-only free medial flap of the second toe by using local anesthesia at the harvest site, and a brachial plexus nerve block was administered for the recipient extremity three days later. This research protocol was approved in advance by the appropriate ethical committee of Sun Yat-sen University and the Long Gang Orthopedics Hospital, and informed consent was provided by all patients. All of our methods were carried out in accordance with approved guidelines.

### Surgical procedures

**Figure 1:**
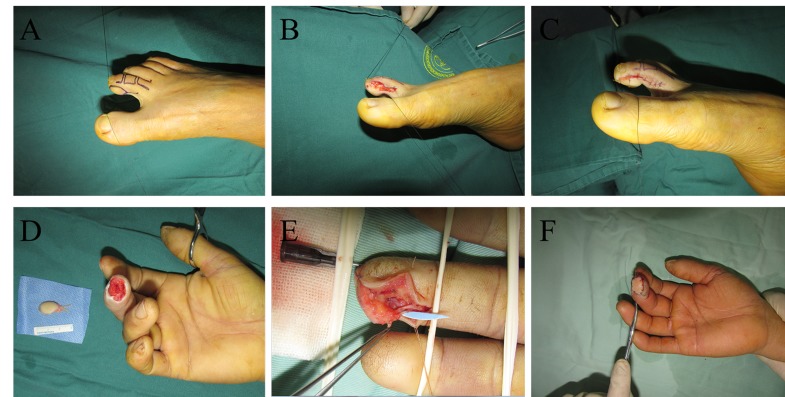
A. Flap design at the donor site before the operation. B. The flap was dissected. C. The donor site was closed without a skin graft. D. The harvested flap and the recipient site before transplantation. E. The anastomosis during transplantation. F. The flap after transplantation.

The second toe flap was designed for the enlarged site of the distal phalanges based on the shape and size of the recipient site as well as the length of the vascular pedicle. First, an incision was made at the flap pedicle according to the designed line to expose the proper arteries and nerves on the medial side of the second toe. When the entry into the flap was confirmed, the branch vessels were ligatured to avoid avulsion. Then, the surrounding skin was cut, the flap was dissected from the deep fascia from the distal portion to the pedicle, and the artery and nerve were also dissected to the proximal or middle phalanges of the second toe. When the flap was only connected with the artery and nerve, the tourniquet was loosened to observe the blood supply of the flap, and then the artery and nerve were cut. Finally, the flap was transplanted to the recipient site; the proximal portion of the recipient site was incised to expose the digital proper artery and nerve. Subsequently, end-to-end anastomosis was performed by using 10-0 Prolene sutures under the assistance of a 10×surgical microscope. One artery and one nerve were anastomosed in these flaps, and the donor sites of the flaps were sutured directly without any skin grafts (Figure 1).

### Postoperative care

**Figure 2:**
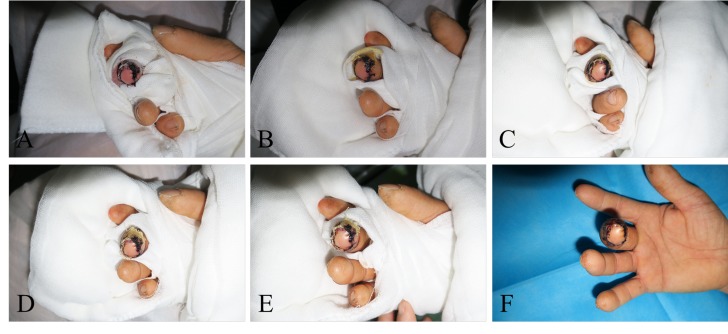
The flap appearance after transplantation on Day 1 (A), Day 3 (B), Day 5 (C), Day 7 (D), Day 9 (E), and Day 11 (F). The blood supply as good without obvious congestion.

The patients were given anti-infection, anticoagulation and antispasmodic therapies after the second-stage surgery. The patients’ blood circulation, skin color, temperature, tension, capillary reactions of the flap and flap congestion were observed and recorded every hour (Figure 2). Cefuroxime was administered for three days, papaverine hydrochloride was administered intramuscularly (30 mg, q6h→qd for day 1→day 7) for seven days, and heparin was continually administered intravenously (12500 IU every 24 hours) for seven days.

### Follow-up assessments

The objective sensory assessments included the Semmes-Weinstein monofilament (SWM) examination and the static 2-point discrimination (s2PD). The Cold Intolerance Symptom Severity (CISS) questionnaire was provided to assess cold intolerance in the patients’ hands. The CISS questionnaire consisted of 6 questions involving feeling coldness and the impact of coldness; each question was measured on a scale from 0 to 10 or 0 to 4, with a minimum total score of 4 (the least severe) and the maximum of 100 (the most severe), where a lower score represents less severe cold intolerance. Scores above 30 were considered to indicate cold intolerance. Additionally, patient satisfaction was then evaluated by using a visual analog scale. Motor function was evaluated by means of fine movements.

### Statistics

Continuous variables are presented as the mean and standard deviation (SD), and the ordinal categorical variables are expressed as frequency and percentage.

## RESULTS

This study included two female patients and six male patients, with an age of 35 (SD 9.8) years old. There were four fingertip injuries on each side, and the injured digits included five middle fingers, two index fingers and one ring finger. According to the Allen classification, of these patients, five patients had Type 3 injuries, and three patients had Type 4 injuries. The reconstruction time was 71.86 (SD 14.75) minutes. The length of the wound was 1.47 cm (SD 0.10), the width of the wound was 0.97 cm (SD 0.23), and the flap sizes ranged from 120 mm^2^ to 180 mm^2^ (Table 1). All eight flaps survived; moreover, leech therapy, continuous bleeding, needle sutures were not utilized in any cases.

**Figure 3:**
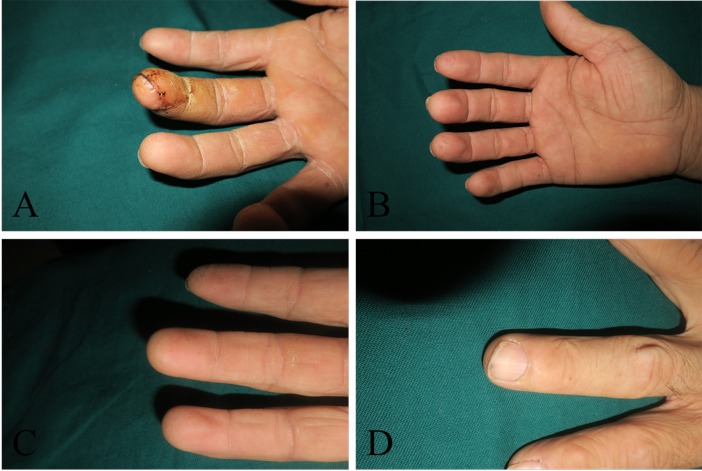
In this patient, the flap appearance 3 weeks (A), 6 months (B), 1 year (C) and 2 years (D) after transplantation. The appearance was satisfactory, and the patient had a normal nail plate.

**Figure 4:**
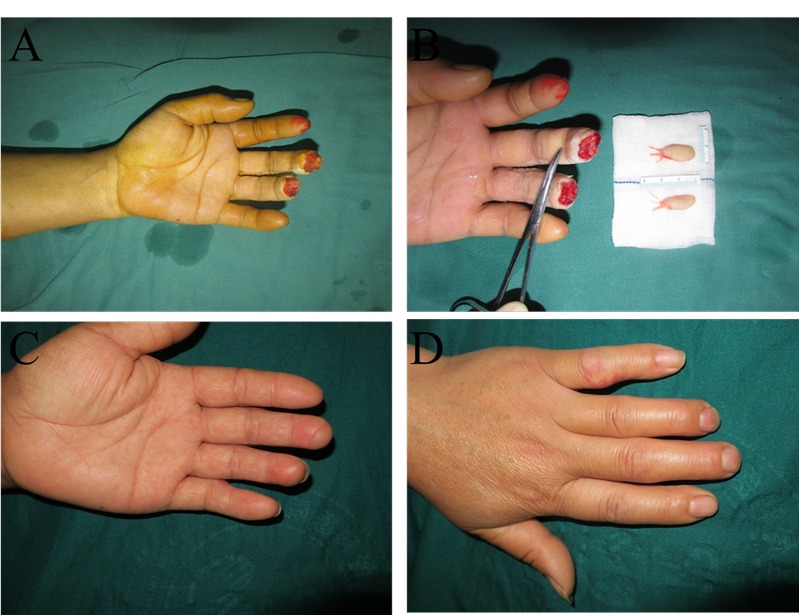
In this patient, the injured fingertips after debridement (A), the flaps before transplantation (B), and the volar (C) and dorsal (D) appearances after 6 months. In this case, the patient injured the left middle and ring fingertips. The middle finger received an artery-only free medial flap of the second toe, while the ring finger received a routine free medial flap of the third toe.

**Figure 5:**
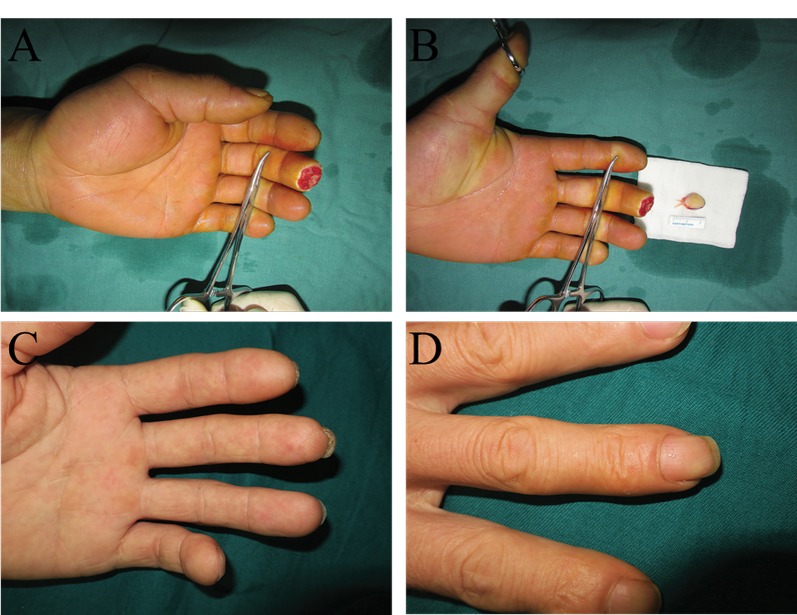
In this patient, the injured fingertip after debridement (A), the flap before transplantation (B), and the volar (C) and dorsal (D) appearances after 9 months.

**Figure 6:**
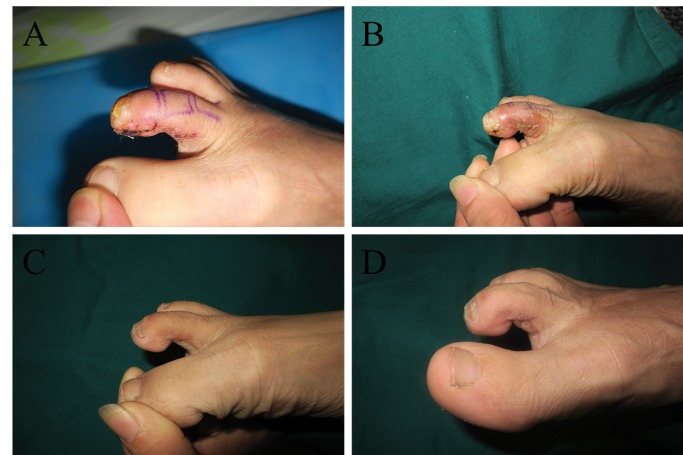
The donor site appearance 11 days (A), 21 days (B), 6 months (C) and 1 year (D) after transplantation. The appearance was satisfactory, and the patient did not have an abnormal gait.

The patients were followed up for 8.6 (SD 2.4) months, and the level of patient satisfaction was 9.5 (SD 0.25). The two-point discrimination value was 4.75 mm (SD 0.89) six months after surgery. The monofilament was 3.00 mm (SD 0.36). Cold intolerance did not appear in five patients, and the other three patients had cold intolerance with grades of 4, 12 and 26 (Table 2). The appearance, pinch movements, and key gripping movements of the finger recovered to almost normal levels (Figure 3, Figure 4, Figure 5 and Video 1), but the range of motion of the injured fingers was not evaluated. Feelings of discomfort did not emerge when the patients were wearing shoes, standing, or walking on the donor toe (Figure 6).

**Video 1:** The patient had satisfied appearance and fine movement. https://doi.org/10.6061/clinics/2019/e1226(Video1).

## DISCUSSION

Approximately one-third of all traumatic injuries impact our hands, and the fingertips are the most commonly injured part of the hands. Fingertip injuries always lead to a reduction in digital function, undesirable shape changes, and psychological burden, which will further affect patients in terms of work and life. Surgical treatment methods aim to close the wound, maximize sensory recovery, preserve finger length, maintain joint function and thus obtain a satisfactory cosmetic appearance (6).

Successful artery-only fingertip reconstruction has been reported sporadically in previous microsurgery studies (4), but this technique has never been adopted for a free-flap transfer. Although this technique could provide us with an accessible method to treat fingertip injuries, surgeons normally opt for a different method when no veins are available for anastomosis. Thus, there is no need to perform vein transplantation and an additional incision to explore recipient or donor veins, which may minimize scarring and decrease the risk of flap survival.

As reported, the utilization of homogeneous flaps in numerous cases has obtained satisfactory recovery results, especially in terms of two-point discrimination (7,8). The flap was chosen from the distal part of the toe to maintain a similar structure and high tactile sensitivity and to enable a precise reconstruction. The flap used for the procedure met the homogeneous standards; therefore, the results from the second toe medial flap transfers in our cases were satisfactory.

The two-point discrimination value was 4.75 mm (SD 0.89) during the follow-up, which was almost equivalent to the value of 4.25 mm (SD 1.5) obtained after the free toe-to-finger flap transfer and was considered as excellent (9). The monofilament was 3.00 (SD 0.36), which was better than that of fingertip replantation with nerve repair (3.34 (SD 0.68)). Cold intolerance did not appear in five patient, and the other three patients had cold intolerance with grades of 4, 12 and 26, indicating that these patients only showed slight discomfort and did not reach a severe degree of cold intolerance. Compared to the high incidence of cold intolerance and poor two-point discrimination values after neurovascular island pedicle flap transfer (9), these results were satisfactory; in addition, the patients’ motor function recovery was also satisfactory. The pinching and key gripping movements were almost at the normal levels with respect to speed and mobility, as seen in Video 1, but quantitative measurements of strength were not assessed, which was a major drawback of this study that will be evaluated in further studies.

The case reports and studies on fingertip replantation and reconstruction are long-standing. Considerable progress in fingertip reconstruction and replantation have been observed since the first case report of successful fingertip replantation; however, as the anastomosis site moves distally (10), the anastomosis of veins has become increasingly challenging due to flaccidity of the vessel wall and the smaller diameter in the distal site. Moreover, vein anastomosis can lead to risky postoperative occlusion, especially when the dorsal veins are severely damaged.

Technically speaking, vein anastomosis cannot be performed at certain distal levels; anastomosis can be considered especially challenging when the diameter is smaller than 0.3 mm (7), which always results in vein insufficiency in the most distal portion. However, paradoxically, vein insufficiency could lead to a significant failure rate in fingertip replantation (10). As a result, artery-only fingertip reconstruction is highly attractive. Some case reports revealed that the most significant factor for amputee survival was to utilize procedures such as arteriovenous anastomosis or artery-to-artery anastomosis to maintain blood flow through other mechanism (5) so that vein repair was not necessary. The fingertip can be salvaged successfully without vein anastomosis through simple surgical or nonsurgical methods such as venous drainage, such as the usage of surgical leeches, bloodletting or early exercise after surgery (4). Hence, the artery-only transfer flap is possible for small flaps. Kakinoki found that the maximum flap size possible without vein anastomosis was 763 mm^2^; the survival rate of larger flaps is significantly lower (11). Our flaps varied from 120 mm^2^ to 180 mm^2^ in size, which provided theoretical evidence for flap survival. As the largest area able to undergo free-flap transplantation without vein anastomosis has not been determined, this area demands further research with animal experiments and anatomical studies. In this regard, this technique is hereby proposed in small flaps (no larger than 180 mm^2^, according this study). In addition, although all the flaps survived without any complications in this study, the artery-only flap is still at high risk of congestion, which may require medical leeches or bloodletting. Hence, observing the circulation of the flap every hour, by referring to the color, temperature and condition of the skin, is advisable.

Patients simultaneously suffering from high blood pressure, diabetes, or vascular disease or those who have a smoking addiction are not suitable for fingertip injuries reconstruction techniques such as flap transfer (3,10,12). In addition, age should be considered a factor because people over 50 years old with severely crushed may be subject to problems such as residual paresthesia and unsatisfactory sensory recovery (12,13). All the patients involved in this study were free of concomitant diseases; therefore, we suggest that this technique be applied to select patients who are generally healthy.

## CONCLUSION

The preliminary data in this study showed that a small artery-only free medial flap transfer of the second toe can lead to satisfactory sensory and motor functions in select cases of fingertip injuries.

## AUTHOR CONTRIBUTIONS

Muwei Li and Ming Huang were responsible for the study conception and design and data acquisition and drafted the article; Yanjun Yang contributed to the data analysis and interpretation; Liqiang Gu revised the article; and Ziqing Zhang, M.D. and Yi Yang were responsible for the study conception and design and data acquisition. All of the authors participated in the surgeries.

## Figures and Tables

**Table 1:** Demographic characteristics of the patients.

Variable	Frequency/mean	Percentage/standard deviation
Sex		
Female	2	25
Male	6	75
Affected hand		
Left	4	50
Right	4	50
Length of wound*	1.47	0.1
Width of wound*	0.97	0.23
Reconstruction time*	71.86	14.75
Age*	35	9.8
BMI*	22.21	3.59
Follow-up period*	8.6	2.4

* Data are presented as the mean ± standard deviation.

**Table 2:** Follow-up results of the patients.

Variable	Case 1	Case 2	Case 3	Case 4	Case 5	Case 6	Case 7	Case 8
Two-point discrimination	4 mm	5 mm	6 mm	4 mm	4 mm	6 mm	5 mm	4 mm
Monofilament	2.83	2.83	3.61	2.83	2.83	2.83	3.61	2.83
CISS score	26	/	/	/	12	4	/	/
